# Increased posterior default mode network activity and structural connectivity in young adult *APOE-*ε4 carriers: a multimodal imaging investigation

**DOI:** 10.1016/j.neurobiolaging.2018.08.026

**Published:** 2019-01

**Authors:** Carl J. Hodgetts, Jonathan P. Shine, Huw Williams, Mark Postans, Rebecca Sims, Julie Williams, Andrew D. Lawrence, Kim S. Graham

**Affiliations:** a Cardiff University Brain Research Imaging Centre, School of Psychology, Cardiff University, Cardiff, Wales, UK; b German Center for Neurodegenerative Diseases (DZNE), Aging and Cognition Research Group, Magdeburg, Germany; c Institute of Psychological Medicine and Clinical Neurosciences, MRC Centre for Neuropsychiatric Genetics and Genomics, Cardiff University, Cardiff, Wales, UK; d Dementia Research Institute, Cardiff University, Cardiff, Wales, UK

**Keywords:** Alzheimer's disease, Default mode network, Diffusion MRI, Medial temporal lobe, Scene processing

## Abstract

Young adult *APOE*-ε4 carriers show increased activity in posterior regions of the default mode network (pDMN), but how this is related to structural connectivity is unknown. Thirty young adults (one half of whom were *APOE*-ε4 carriers; mean age 20 years) were scanned using both diffusion and functional magnetic resonance imaging. The parahippocampal cingulum bundle (PHCB)—which links the pDMN and the medial temporal lobe—was manually delineated in individual participants using deterministic tractography. Measures of tract microstructure (mean diffusivity and fractional anisotropy) were then extracted from these tract delineations. *APOE*-ε4 carriers had lower mean diffusivity and higher fractional anisotropy relative to noncarriers in PHCB, but not in a control tract (the inferior longitudinal fasciculus). Furthermore, PHCB microstructure was selectively associated with pDMN (and medial temporal lobe) activity during a scene discrimination task known to be sensitive to Alzheimer's disease. These findings are consistent with a lifespan view of Alzheimer's disease risk, where early-life, connectivity-related changes in specific, vulnerable “hubs” (e.g., pDMN) lead to increased neural activity. Critically, such changes may reflect reduced network efficiency/flexibility in *APOE*-ε4 carriers, which in itself may portend a faster decline in connectivity over the lifespan and ultimately trigger early amyloid-β deposition in later life.

## 1 Background

The default mode network (DMN) is a large-scale brain system displaying continuously high levels of coordinated activity in the resting state (Raichle, 2015). Rather than constituting a single, unitary brain network, the DMN can be divided into several functionally dissociable subsystems (Andrews-Hanna et al., 2010, Raichle, 2015), which are differentially vulnerable to Alzheimer's disease (AD) pathology (Myers et al., 2014). Notably, the posterior DMN (pDMN), comprising posterior cingulate, precuneus, and retrosplenial cortex (Cauda et al., 2010), is one of the earliest brain areas to undergo amyloid-β (Aβ) accumulation and reduced metabolism in AD (Gonneaud et al., 2016, Palmqvist et al., 2017).

The pDMN has been labeled the brain's structural “hub” given its dense functional and structural interconnectivity and associated high levels of baseline activity/metabolism (Bero et al., 2012, Buckner et al., 2009, Hagmann et al., 2008). Based on its extensive connectivity with the medial temporal lobe (MTL) (Greicius et al., 2009, Heilbronner and Haber, 2014), it has been proposed that the pDMN anchors a posteromedial system involved in forming mental scenes, or “situation models” (Murray et al., 2016, Ranganath and Ritchey, 2012). Critically, such models may underpin a range of cognitive processes that are affected in early AD, including episodic past/future thinking (Addis et al., 2009, Irish et al., 2015), spatial navigation (Lester et al., 2017), and complex scene discrimination (Lee et al., 2006).

The topographical overlap between the pDMN and regions showing early Aβ accumulation has led to a “lifespan systems vulnerability” (LSV) account of AD, in which lifespan increases in activity and connectivity predispose regions to Aβ deposition (Buckner et al., 2009, de Haan et al., 2012, Jagust and Mormino, 2012). Consistent with this, a transgenic mice study reported that interstitial Aβ levels were associated with increased markers of neural activity, and this, in turn, predicted Aβ deposition in the pDMN (Bero et al., 2011; see also Yamamoto et al., 2015). A further study showed that region-specific levels of functional connectivity in young Aβ-mice were proportional to the degree of Aβ burden in older animals (Bero et al., 2012).

Human neuroimaging studies have reported similar within-subject associations between the degree of baseline pDMN functional connectivity and subsequent Aβ load in both mild cognitive impairment (MCI) (Myers et al., 2014) and cognitively normal older adults (Jack and Holtzman, 2013). Although these findings suggest that functional changes may drive pathology, they could also reflect a compensatory response induced by early Aβ burden in key networks (Jagust and Mormino, 2012, Jones et al., 2016). Given the hypothesis that later-life Aβ deposition in the pDMN arises from increased functional activity/connectivity across the lifespan, a key prediction is that alterations in brain structure and/or function may also be evident in young at-risk individuals who are highly unlikely to harbor Aβ (de Haan et al., 2012, Mormino, 2014).

The *APOE*-ε4 allele is the strongest genetic risk factor for both sporadic early- and late-onset AD (Liu et al., 2013) and is strongly linked to later-life Aβ accumulation (Gonneaud et al., 2016). Functional magnetic resonance imaging (fMRI) studies in young adults have found typically that *APOE*-ε4 carriers show increased activity, relative to noncarriers, in the pDMN and interconnected MTL regions, particularly during episodic memory paradigms (Dennis et al., 2010, Filippini et al., 2009). *APOE*-ε4 carriers have also been shown to have greater intrinsic functional connectivity in the DMN during “rest” (Filippini et al., 2009). Our recent study found that young *APOE*-ε4 carriers show increased functional activity in the pDMN, relative to noncarriers, during a perceptual discrimination task for scenes, but not faces or objects (Shine et al., 2015)—consistent with scene-specific deficits reported in early AD (Lee et al., 2006).

Given the view that pDMN vulnerability to Aβ accumulation is linked to its role as a large-scale connectivity hub (Jagust and Mormino, 2012, Jones et al., 2016), heightened pDMN activity in young *APOE*-ε4 carriers may itself be linked to variation in structural connectivity (Brown et al., 2011, de Haan et al., 2012), particularly with other regions affected early in AD. The major white matter connection linking the pDMN with MTL (particularly parahippocampal gyrus [PHG]; Greicius et al., 2009, Heilbronner and Haber, 2014, Mufson and Pandya, 1984) is the parahippocampal cingulum bundle (PHCB). Studies applying diffusion MRI (dMRI)—a method allowing in vivo quantification of white matter microstructure—have reported greater mean diffusivity (MD) and lower fractional anisotropy (FA) in the PHCB of cognitively normal older *APOE*-ε4 carriers compared with noncarriers (Heise et al., 2014). One cross-sectional dMRI study found that young adult *APOE*-ε4 carriers had higher PHCB FA than noncarriers but showed a steeper decline across life, leading to a relative reduction in FA from midlife onward (Felsky and Voineskos, 2013; see also Brown et al., 2011). Disruption of this pathway is also seen in both MCI and AD (Mito et al., 2018, Rieckmann et al., 2016) and has been linked to pDMN activity/metabolism in AD (Villain et al., 2008) and Aβ burden in preclinical AD (Racine et al., 2014). Overall, these studies suggest an early-life vulnerability of a broader posterior network that is structurally underpinned by the PHCB (Ranganath and Ritchey, 2012).

It is unclear, however, whether these PHCB microstructural alterations are evident earlier in life, concomitant with the identified functional changes in college-aged adults (Dennis et al., 2010, Filippini et al., 2009, Shine et al., 2015). Moreover, if increased activity in pDMN stems from its role as a large-scale connectivity hub (Buckner et al., 2009), then those individuals who show elevated pDMN activity (Filippini et al., 2009, Shine et al., 2015) should also have “increased” structural connectivity (de Haan et al., 2012). To address these questions, we applied high–angular resolution dMRI (HARDI; Tuch et al., 2002), alongside constrained spherical deconvolution (CSD) tractography (Jeurissen et al., 2011), to test whether the presence of an *APOE*-ε4 allele in young adults, who are unlikely to harbor amyloid burden (Mormino, 2014), influences PHCB tissue microstructure. Given evidence that young *APOE*-ε4 carriers show elevated pDMN activity at rest (Filippini et al., 2009) and during tasks (Shine et al., 2015), we predicted that *APOE*-ε4 carriers would show greater FA and lower MD in the PHCB, compared with noncarriers, but not in a control tract (the inferior longitudinal fasciculus [ILF]). Finally, to demonstrate a link between activity and connectivity, as predicted by an LSV view of AD risk, we examined whether interindividual variation in PHCB tissue microstructure was associated with pDMN activity during a scene discrimination task that is sensitive to early AD (Lee et al., 2006).

## 2 Material and methods

### 2.1 Participants

A total of 125 psychology undergraduates provided a saliva sample for DNA extraction. Genotyping was performed using Applied Biosystems (Applied Biosystems, Foster City, CA, USA), Assay-on-demand TaqMan SNP Genotyping Assays, C_3084793_20 and C_904973_10 corresponding to *APOE* single nucleotide polymorphisms (SNPs) rs429358 and rs7412, respectively, and run on an LJL Biosystems Analyst HTS Assay Detection Platform (LJL Biosystems, Sunnyvale, CA, USA). Haplotypes corresponding to *APOE*-ε2, ε3, and ε4 were then deduced. Genotyping was successful in 100/125 participants. The genotypic distribution for those 100 participants was ε2ε2 (1/100, 1%), ε2ε3 (10/100, 10%), ε2ε4 (1/100, 1%), ε3ε3 (69/100, 69%), ε3ε4 (19/100, 19%), and ε4ε4 (0/94, 0%), thus closely matching the expected frequencies in the normal population (χ^2^ = 4.48, *df* = 5, *p* = 0.48) (Menzel et al., 2015). Based on the presence/absence of an *APOE*-ε4 allele, two groups (20 participants per group) were created, pairwise matched for gender, educational level, and age. Owing to scanning nonattendance, MRI contraindications, and withdrawal during testing, the available sample for the reported analysis was 30 participants (15 per group; 14 females per group)—a sample size similar to other structural/functional studies of *APOE*-ε4 (Dennis et al., 2010, Filippini et al., 2009, Oh and Jagust, 2013). The noncarrier *APOE* allele distribution was 10 *APOE*-ε3ε3 and 5 *APOE*-ε2ε3 individuals. The carrier *APOE* allele distribution was 14 *APOE*-ε3ε4 and 1 *APOE*-ε2ε4. Both groups were matched for age (carriers: 19.7 years, SD = 0.84; noncarriers: 19.7 years, SD = 0.89) and education level. Family history of neurodegenerative disease was matched across the groups, with two reports of a positive family history in each group, as assessed using a self-report family history questionnaire. In the *APOE*-ε4 noncarrier group, there was one report of Parkinson's disease (in a great uncle aged approximately 75 years) and one report of dementia (variety not known) in a grandmother (in her 60s). In the *APOE*-ε4 carrier group, there was also a report of Parkinson's disease in a grandmother (in her late 50s) and one report of dementia (variety not known) in a great grandmother (aged 90 years).

All participants were right-handed, native English speakers with normal or corrected-to-normal vision and had no self-reported history of neuropsychiatric disorders or substance abuse, as confirmed using the Mini-International Neuropsychiatric Interview (conducted by CJH; Sheehan, 1998). All experimental procedures were conducted in accordance with, and were approved by, the Cardiff University School of Psychology Research Ethics Committee. Informed consent was obtained from all participants, and research was conducted in a double-blind manner.

### 2.2 MRI scan parameters

Imaging data were collected at the Cardiff University Brain Research Imaging Center (CUBRIC) using a GE 3-T HDx MRI system (General Electric Healthcare, Milwaukee, WI, USA) with an 8-channel receive-only head coil. Whole-brain HARDI (Tuch et al., 2002) data were acquired using a diffusion-weighted single-shot spin-echo echo-planar imaging (EPI) pulse sequence with the following parameters: TE = 87 ms; voxel dimensions = 2.4 × 2.4 × 2.4 mm^3^; field of view (FOV) = 23 × 23 cm^2^; 96 × 96 acquisition matrix; 60 slices (oblique axial with 2.4 mm thickness). Acquisitions were cardiac gated using a peripheral pulse oximeter (Habib et al., 2010). Gradients were applied along 30 isotropic directions with b = 1200 s/mm^2^. Three nondiffusion-weighted images were acquired with b = 0 s/mm^2^. For task-fMRI, whole-brain blood-oxygen level-dependent (BOLD) EPI data were acquired with the following parameters: TR/TE = 3000/35 ms; FOV = 240 mm; 64 × 64 acquisition matrix; 90° flip angle; ASSET (acceleration factor); 42 slices (interleaved). Each slice was 2.4-mm thick with a 1-mm interslice gap (3.4 × 3.4 × 2.4 mm voxels). Slices were acquired with a 30° axial-to-coronal tilt relative to the AC-PC line (anterior upward). The first 4 volumes of each scanning run were discarded to allow for signal equilibrium. A field map was acquired to improve registration and reduce image distortion as a result of magnetic field inhomogeneity (TR = 20 ms, TE = 7 ms/9 ms, FOV = 384 × 192 × 210 mm, 128 × 64 × 70 acquisition matrix, 10° flip angle). The field map used the same slice orientation as the EPI data. Additional high-resolution anatomical images were acquired using a T1-weighted 3D FSPGR sequence: TR/TE = 7.8/3.0 seconds; FOV = 256 × 256 × 176 mm; 256 × 256 × 176 acquisition matrix; 20° flip angle; 178 axial slices; 1 mm isotropic resolution.

### 2.3 Diffusion MRI

#### 2.3.1 Preprocessing

Motion and eddy current correction was conducted using ExploreDTI (Leemans and Jones, 2009). Partial volume-corrected maps of tissue FA and MD were generated by applying the bi-tensor free water elimination (FWE) procedure (Pasternak et al., 2009). These partial volume-corrected maps were used in the subsequent dMRI analyses (see Section 2.4 and 3). MD (10^−3^ mm^2^ s^−1^) reflects a combined average of axial (diffusion along the principal axis) and radial diffusion (diffusion along the orthogonal direction). FA reflects the extent to which diffusion is anisotropic, or constrained along a single axis, and can range from 0 (fully isotropic) to 1 (fully anisotropic).

#### 2.3.2 Tractography

Deterministic whole-brain tractography was conducted in ExploreDTI (Leemans and Jones, 2009) using the CSD model (Jeurissen et al., 2011), which extracts multiple peaks in the fiber orientation density function (Vettel et al., 2017). Streamlines were reconstructed using the following parameters: fiber orientation density function amplitude threshold = 0.1; step size = 0.5 mm; and angle threshold = 60°.

**Fig. 1:**
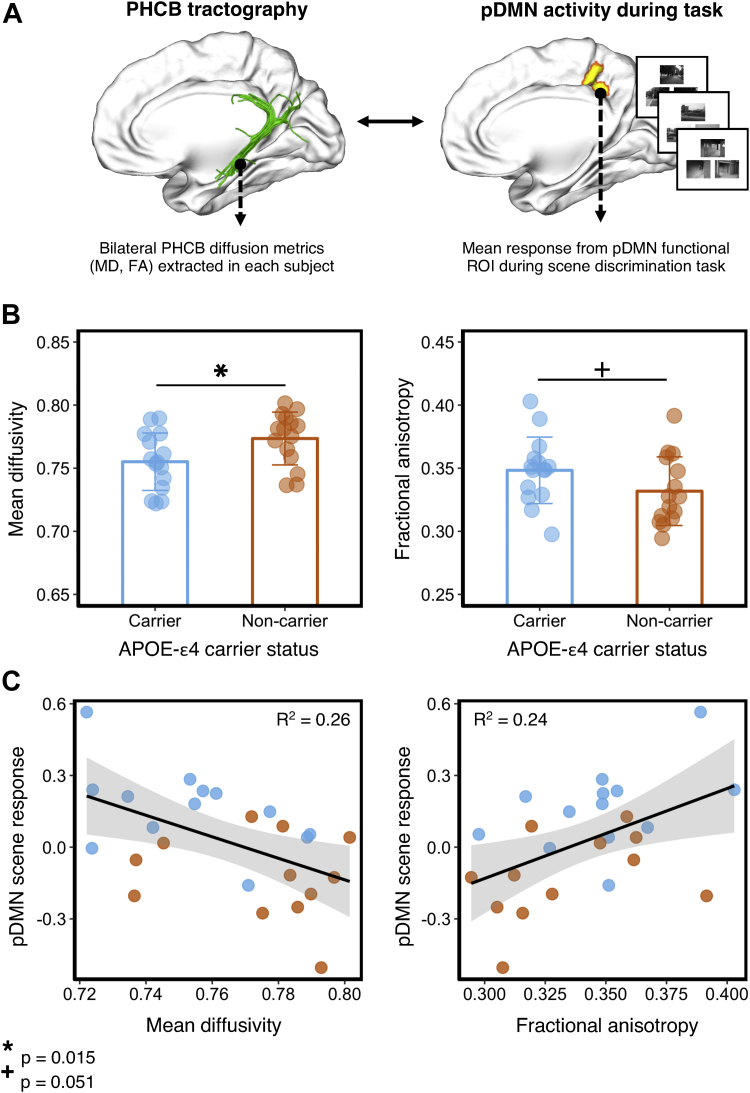
Comparing PHCB tissue microstructure between *APOE*-ε4 carriers and noncarriers. (A) Left: Deterministic tractography was conducted in each participant, and free water–corrected indices of bilateral PHCB microstructure (MD, FA) were extracted. Right: To examine associations with functional activity, these metrics were correlated with BOLD activity from an independently defined pDMN functional ROI during a perceptual discrimination task (Shine et al., 2015). Example scene trials for the perceptual “odd-one-out” discrimination task are shown. (B) Plots comparing mean bilateral PHCB MD and FA for *APOE*-ε4 carriers and noncarriers. Individual data points are displayed jittered on each bar. (C) Scatter plots showing the association between scene (vs. “size” baseline) activity in the pDMN and MD (left) and FA (right) in the PHCB. A total of 25 data points are shown on each scatter plot (13 carriers, blue markers; 12 noncarriers, orange markers; see Section 3.4). Abbreviations: BOLD, blood-oxygen level dependent; FA, fractional anisotropy; MD, mean diffusivity; PHCB, parahippocampal cingulum bundle; pDMN, posterior default mode network; ROI, region of interest. (For interpretation of the references to color in this figure legend, the reader is referred to the Web version of this article.)

Three-dimensional reconstructions of the PHCB (Fig. 1A) were obtained from individual participants using a Boolean, way-point region-of-interest (ROI) approach, where “AND” and “NOT” ROIs were applied and combined to isolate PHCB streamlines in each participant's whole-brain tractography data. These ROIs were drawn manually on the direction-encoded FA maps in native space by one experimenter (HW) who was blind to *APOE*-ε4 carrier status and quality-assessed by a second experimenter (CJH).

##### 2.3.2.1 Parahippocampal cingulum reconstruction

Reconstruction of the PHCB followed a previously published and reliable protocol [termed “restricted parahippocampal cingulum”; see (Jones et al., 2013a, Jones et al., 2013b)]. The first step was to identify the corpus callosum on the mid-sagittal slice. This was followed by identifying the parasagittal slice that afforded the most extensive view of the cingulum bundle along its long (anterior-to-posterior) axis. The corpus callosum on this plane provided key landmarks for ROI placement in each hemisphere. The initial seed ROI (from where tractography is initiated) was positioned by (1) locating the most inferior part of the splenium (i.e., the posterior bend of the corpus callosum) and (2) placing an AND ROI on the axial plane 3 to 4 slices superior to this. The second AND ROI was placed three slices inferior to this. For the NOT ROI, the anterior-posterior midpoint of the corpus callosum was first identified. This was defined as the midpoint between the most posterior part at the flexure of the splenium and the most anterior part of the genu (i.e., the anterior bend). The NOT ROI was then positioned 5 slices posterior to this midpoint. Following initial tract reconstruction, erroneous streamlines were removed using additional NOT ROIs.

##### 2.3.2.2 ILF reconstruction (control tract)

Fiber tracking of the ILF was performed using a two-ROI approach in each hemisphere (Wakana et al., 2007). First, the posterior edge of the cingulum bundle was identified on the sagittal plane. Reverting to a coronal plane at this position, a SEED ROI was placed that encompassed the whole hemisphere. To isolate streamlines extending towards the anterior temporal lobe, a second ROI was drawn at the most posterior coronal slice in which the temporal lobe was not connected to the frontal lobe. Here, an additional AND ROI was drawn around the entire temporal lobe. Similar to the aforementioned PHCB protocol, any anatomically implausible streamlines were removed using additional NOT ROIs. This approach was carried out in both hemispheres.

#### 2.3.3 Analysis of tractography data

Following bilateral tract reconstruction for both tracts (PHCB and ILF), the partial volume-corrected maps for FA and MD were intersected with the PHCB and ILF tract masks to obtain mean bilateral measures of tract microstructure (MD and FA). Partial volume-corrected MD and FA values in *APOE*-ε4 carriers and noncarriers were compared directly using directional Welch *t*-tests in R. We also report default JZS Bayes factors for our key analyses, computed using JASP (https://jasp-stats.org). The Bayes factor (expressed as BF_10_) indicates the strength of evidence that the data provide for the alternative hypothesis (H1) relative the null hypothesis (H0). A BF_10_ much greater than 1 allows us to conclude that there is substantial evidence for the alternative versus the null hypothesis (Wagenmakers et al., 2017).

#### 2.3.4 Tract-based spatial statistics

Voxelwise statistical analysis of the dMRI data was carried out using tract-based spatial statistics (TBSS) (Smith et al., 2006). This method involved nonlinearly projecting participants' partial volume-corrected statistical maps (both MD and FA) onto a mean tract skeleton and then applying voxelwise cross-subject statistics. We applied a general linear model contrasting *APOE*-ε4 carriers and noncarriers for each dMRI metric. To restrict our analysis to the PHCB, we extracted the PHCB mask [labeled “cingulum (hippocampus)”] from the Johns Hopkins University ICBM-DTI-81 white-matter tractography atlas using FSLView (for similar approach, see Heise et al., 2014). Significant clusters were extracted using threshold-free cluster enhancement (TFCE; Smith and Nichols, 2009) with a corrected alpha of *p* = 0.05. Additional exploratory whole-brain analyses were conducted using the same TFCE-corrected statistical threshold. All reported coordinates are in Montreal Neurological Institute (MNI-152) space.

### 2.4 Functional MRI

#### 2.4.1 Preprocessing

Functional MRI preprocessing was performed using FSL (www.fmrib.ox.ac.uk/fsl) (Jenkinson et al., 2012) and involved motion correction using MCFLIRT (Jenkinson et al., 2002), brain extraction using BET (Smith, 2002), field map unwarping using FUGUE (Jenkinson et al., 2002), spatial smoothing with a gaussian kernel of full-width half-maximum 5 mm, mean-based intensity normalisation, and high-pass temporal filtering (gaussian-weighted least-squares straight line fitting, with sigma = 100 seconds). Time-series analysis was performed using FMRIB's Improved Linear Model with local autocorrelation correction. Registration to high-resolution anatomical scans (per participant) and to the standard Montreal Neurological Institute (MNI-152) template image was carried out using FLIRT. Following preprocessing, analyses were conducted at the single-subject level using FMRIB's Improved Linear Model.

#### 2.4.2 Participant exclusion criteria

Five participants were excluded from the fMRI analysis sample due to participant motion (4 participants) and scanner error (1 participant) resulting in a final sample of 25 participants (13 carriers and 12 noncarriers). For the fMRI sample, the *APOE*-ε4 carrier allele distribution was 12 *APOE*-ε3ε4 and 1 *APOE*-ε2ε4, and the noncarrier allele distribution was 8 *APOE*-ε3ε3 and 3 *APOE*-ε2ε3 individuals.

#### 2.4.3 Odd-one-out fMRI paradigm

In this task, participants were presented with three stimuli on each trial (top center, bottom left, and bottom right) and instructed to select the “odd-one-out” as quickly and as accurately as possible. Here, we analysed scene and face odd-one-out trials, with a “size” odd-one-out condition acting as a baseline condition. The scene stimuli comprising each trial were naturalistic photographs of outdoor urban and rural environments, taken from a normal eye-level observer perspective. On each trial, participants viewed two images of a single locale from different viewpoints and one different locale (i.e., the “odd-one-out”). Face stimuli were grayscale photographs of human faces (half female/half male) and were overlaid on a black background (170 × 216 pixels). Similar to the scene condition, two faces were the same individual presented from different viewpoints and the target was a different face presented from a different viewpoint. For the size baseline task, three black squares were presented. On each trial, two of the squares were identical in size and a third square was either slightly larger or smaller. The difference in length between target and nontargets could vary between 9 and 15 pixels. The position of the squares on the screen was jittered, so that none of the edges lined up along vertical or horizontal axes. All stimuli were trial-unique (i.e., never repeated once shown in the task).

Each trial was presented for 6 seconds with a jittered intertrial interval of 500–4000 ms. The task was administered in the scanner over three functional imaging runs. Within each run, trials for a given category were presented in mini-blocks of three successive trials. The order in which category “triplets” were presented was counterbalanced across participants. Overall, 18 trials were presented per category per run resulting in 54 trials per condition overall. An equal number of targets appeared at each screen position (i.e., top center, bottom left, and bottom right) within each stimulus condition. Stimuli were presented in the scanner using ePrime (Psychology Software Tools, Inc, Sharpsburg, PA, USA) and projected onto the screen behind the participant using a Canon SX60 LCOS projector system combined with the Navitar SST300 zoom converter lens. Button responses in the scanner were acquired using a right-hand MR compatible button box.

#### 2.4.4 Functional MRI analysis

In this study, our primary fMRI measure was the mean BOLD response (percent signal change) in the pDMN for scene and face trials (Fig. 1A). To extract these, a general linear model was implemented to examine the BOLD response associated with correct scene and face trials relative to “size” baseline. The duration of the regressors corresponded to the trial duration (i.e., 6 seconds) relative to trial onset, and the BOLD signal was modeled using a standard hemodynamic response function. The three fMRI runs for each participant were concatenated using a fixed-effects model in FEAT and co-registered to the MNI152 2-mm template. To avoid “double-dipping” when extracting the response for each category (Kriegeskorte et al., 2010), the pDMN functional ROI was defined using an independent task (i.e., 1-back working memory; see Shine et al., 2015); this independently defined ROI reflects a significant group difference (carriers > noncarriers) within the right pDMN during scene working memory. This unilateral functional ROI is freely available at the following NeuroVault URL: https://neurovault.org/collections/4048/.

The parameter estimates for scene and face trials (vs. “size” baseline) were extracted from the pDMN functional ROI and converted to percent signal change using the Featquery tool in FSL. These measures, calculated in each participant separately, were then correlated with our key diffusion metrics using directional Pearson's r correlations. Directional Bayes factors and 95% Bayesian credibility intervals (BCIs) are reported for all correlations. BCIs inform us that, given our observed data, there is a 95% probability that the true value of our effect (Pearson's r) lies within this interval (Dienes, 2014). To test whether associations between PHCB tract microstructure and pDMN activity were greater for navigationally relevant scenes (vs. faces), as predicted by models of pDMN function (Murray et al., 2016, Ranganath and Ritchey, 2012), we directly compared individual coefficients using the Steiger Z test (one-tailed) of dependent correlations, as implemented using “cocor” (http://comparingcorrelations.org/) (Diedenhofen and Musch, 2015).

## 3 Results

### 3.1 Comparing PHCB microstructure using tractography

*APOE*-ε4 allele carriers had significantly lower MD compared with noncarriers [t (28) = 2.3 *p* = 0.015, d = 0.84, BF_10_ = 4.55; Fig. 1B]. Although there was a strong trend for PHCB FA in the predicted direction, the between-group difference just failed to reach significance [t (28) = 1.69, *p* = 0.051, d = 0.62, BF_10_ = 1.83; Fig. 1B].

Given that *APOE*-ε4 is reported to have a stronger effect on AD-relevant neuroimaging marker in females (Heise et al., 2014, Ungar et al., 2014), we also conducted this analysis with males removed (one individual from each group). A significant difference was found between carriers and noncarriers for PHCB MD, though with a slightly larger effect size [t (26) = 2.42, *p* = 0.012, d = 0.92, BF_10_ = 5.5]. A significant difference was also found for PHCB FA [t (26) = 2, *p* = 0.03, d = 0.75, BF_10_ = 2.82].

Furthermore, based on studies reporting a protective effect of the *APOE*-ε2 allele on AD biomarkers (Kim et al., 2017), we compared PHCB microstructural measures with the *APOE*-ε2 carriers removed from the sample (5 from the *APOE*-ε4 noncarrier group, 1 from the *APOE*-ε4 carrier group). With these individuals removed, a significant difference between carriers and noncarriers for PHCB MD was still observed [t (22) = 1.97, *p* = 0.03, d = 0.82, BF_10_ = 2.73]. No difference between carriers and noncarriers was found for PHCB FA [t (26) = 1.18, *p* = 0.13, d = 0.49, BF_10_ = 1.04].

### 3.2 Control tract

As a control tract, we compared tract microstructure between carriers and noncarriers for the ILF. The ILF is a ventral, temporo-occipital association tract implicated in semantic cognition (Hodgetts et al., 2017), which, conversely to the PHCB, is less affected by AD than by semantic dementia (Bejanin et al., 2017). There were no significant differences between *APOE*-ε4 carriers and noncarriers for either MD [t (28) = 0.58 *p* = 0.29, d = 0.21; BF_01_ = 1.86] or FA [t (28) = 1.05, *p* = 0.15, d = 0.38; BF_01_ = 1.18]. Again, to examine whether the presence of a male in each group masks possible differences between carriers and noncarriers, we compared ILF microstructure for the females only. As above, no significant differences were found for either measure of ILF microstructure [MD: t (28) = 0.33, *p* = 0.37, d = 0.12, BF_01_ = 2.23; FA: t (28) = 1.33, *p* = 0.1, d = 0.5, BF_10_ = 1.18]. Similarly, removing carriers of a potentially protective allele, *APOE*-ε2, did not alter these findings (all *p*-values > 0.31).

### 3.3 Voxelwise approach

**Fig. 2:**
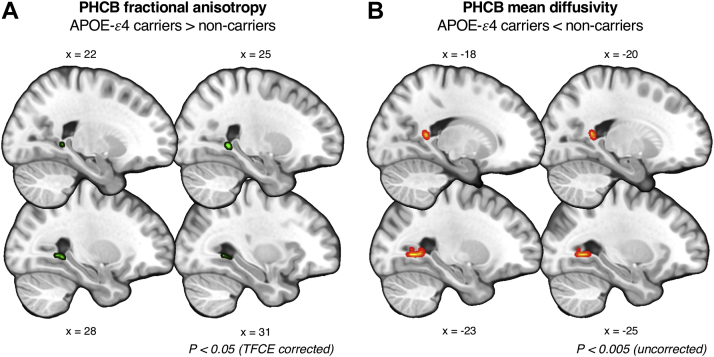
Comparing parahippocampal cingulum bundle (PHCB) microstructure in *APOE*-ε4 carriers and noncarriers using TBSS. (A) A significant cluster (shown in green) was found showing greater FA in *APOE*-ε4 carriers versus noncarriers in the posterior PHCB (*p* < 0.05, TFCE-corrected). (B) A subthreshold cluster (shown in red-yellow) reflecting lower MD in *APOE*-ε4 carriers versus noncarriers was identified in the posterior PHCB (*p* < 0.005, uncorrected). For visualization purposes, clusters have been “thickened” using “TBSS fill” in FSL. There were no voxelwise differences for MD that survived stringent correction. Abbreviations: FA, fractional anisotropy; MD, mean diffusivity; PHCB, parahippocampal cingulum bundle; TBSS, tract-based spatial statistics. (For interpretation of the references to color in this figure legend, the reader is referred to the Web version of this article.)

TBSS analyses identified a significant cluster in the right posterior PHCB for FA (*p* = 0.02; 29, −49, −1), reflecting higher FA in *APOE*-ε4 carriers (Fig. 2)—consistent with the tractography analysis. We found no TFCE-corrected clusters for MD. Using an uncorrected threshold of *p* = 0.005 (Postans et al., 2014), we identified a significant cluster in the left posterior PHCB reflecting lower MD in carriers (*p* < 0.001; −28, −58, 0). An exploratory whole-brain analysis (TFCE-corrected) revealed no significant clusters for either metric.

### 3.4 The relationship between pDMN activity and PHCB microstructure

To examine the functional relevance of these structural connectivity metrics, we tested whether interindividual variation in PHCB microstructure (MD and FA) was associated with fMRI response in the pDMN (Section 2.4.3) during an “odd-one-out” discrimination task for scenes and faces (Shine et al., 2015). Across all individuals, we found a significant negative association between PHCB MD and scene activity (vs. “size” baseline) in the pDMN (r = −0.5, *p* < 0.01, BF_−0_ = 12.1, 95% BCI [−0.73, −0.13]; Fig. 1C). There was no significant association between MD and face activity (r = −0.03, *p* = 0.01, BF_−0_ = 0.29, 95% BCI [−0.45, −0.01]). A one-tailed Steiger Z test revealed a significant difference between these coefficients (z = 2.5, *p* < 0.01). For PHCB FA, we likewise observed a significant association with scene, but not face, pDMN BOLD response (scene: r = 0.49, *p* < 0.01, BF_+0_ = 8.87, 95% BCI [0.12, 0.72]); face: r = 0.12, *p* = 0.29, BF_+0_ = 0.41, 95% BCI [−0.45, −0.01]; Fig. 1C). The correlation between PHCB FA and scene activity was significantly greater than the correlation with face activity (z = 2, *p* = 0.02).

### 3.5 The relationship between MTL activity and PHCB microstructure

**Fig. 3:**
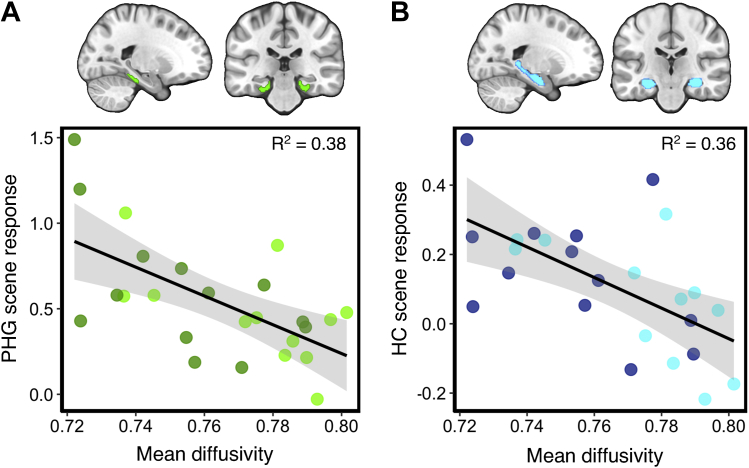
Correlations between scene activity in the medial temporal lobe and parahippocampal cingulum bundle (PHCB) microstructure. Scatter plots showing the association between PHCB mean diffusivity and scene (vs. “size” baseline) activity in the (A) posterior parahippocampal gyrus (PHG, green markers) and (B) hippocampus (blue markers). A total of 25 data points are shown on each scatter plot (13 carriers, dark markers; 12 noncarriers, light markers; see Section 3.5).

To examine structure-function associations within interconnected MTL regions, we correlated PHCB microstructural measures and scene-sensitive BOLD activity within two MTL regions linked to the pDMN via the PHCB—the posterior PHG and hippocampus (Bubb et al., 2018, Mufson and Pandya, 1984). Bilateral ROIs of the posterior PHG and hippocampus were created using probabilistic masks from the Harvard-Oxford cortical and subcortical atlases in FSL. A probability threshold of 50% was applied to restrict these to gray matter. We found a strong significant association between scene-sensitive BOLD response in the posterior PHG and PHCB MD (r = −0.62, *p* < 0.001, BF_−0_ = 84.5; Fig. 3A). A weaker, albeit significant, association was found between PHG BOLD and PHCB FA (r = 0.34, *p* = 0.05, BF_+0_ = 1.7). For the hippocampus, a similarly strong negative correlation was likewise observed between scene-sensitive activity and MD (r = −0.6, *p* < 0.001, BF_−0_ = 61.5; Fig. 3B). A significant association was also found for PHCB FA (r = 0.34, *p* = 0.04, BF_+0_ = 2.1).

### 3.6 Odd-one-out behavioral performance

Finally, to examine any potential behavioral differences in the odd-one-out task, mean accuracy (proportion correct) and response time were each submitted to a two-way mixed-model ANOVA including a between-subject factor of the *APOE* group (*APOE*-ε4 carriers, *APOE*-ε4 noncarriers) and a within-subject factor of category (scenes, faces). There was no significant effect of group [*F*(1, 28) = 0.05, *p* = 0.82; BF_01_ = 3.03] or category [*F*(1, 28) = 2.5, *p* = 0.12, BF_01_ = 1.18] on task accuracy, and no significant interaction between these factors [*F*(1, 28) = 0.2, *p* = 0.66, BF_01_ = 9.3]. Similarly, response times were matched across *APOE* groups, and there was no interaction with item category (all *p*-values > 0.32). There was a main effect of stimulus category [*F*(1, 28) = 8.86, *p* < 0.01, BF_10_ = 4.1), reflecting significantly faster response times for faces versus scenes [t(29) = 3, *p* < 0.01, BF_10_ = 7.12].

## 4 General discussion

Based on the view that pDMN vulnerability to Aβ arises from its role as a large-scale connectivity hub (Buckner et al., 2009, de Haan et al., 2012), we asked whether young adults at heightened genetic risk for AD (via presence of the *APOE*-ε4 allele) would show increased pDMN structural connectivity (Greicius et al., 2009). Supporting this hypothesis, we found that *APOE*-ε4 carriers, relative to noncarriers, had microstructural differences in the PHCB—a white matter tract linking the pDMN with the MTL, particularly parahippocampal regions (Heilbronner and Haber, 2014). Moreover, interindividual variation in PHCB microstructure was selectively associated with pDMN (and MTL) activity during a scene discrimination task that is sensitive to early AD (Lee et al., 2006).

The pDMN has been labeled the brain's epicenter (Hagmann et al., 2008), given its disproportionately high structural and resting-state functional connectivity (Buckner et al., 2009, Hagmann et al., 2008) and metabolism (Oh et al., 2016). This region is also one of the first brain areas to undergo Aβ deposition in AD (Gonneaud et al., 2016, Palmqvist et al., 2017). The early deposition of Aβ in the pDMN suggests that the high processing demands on this region may, over the lifespan, lead to Aβ accumulation and ultimately network failure and cognitive decline (Bero et al., 2011, Jones et al., 2016). In human neuroimaging studies, strong within-subject correspondence has been found between pDMN functional connectivity strength and subsequent Aβ load in individuals with MCI (Myers et al., 2014). Elevated pDMN connectivity in low-amyloid individuals (Aβ−) has also been associated with increased Aβ deposition at follow-up (Jack et al., 2013). These increases in functional connectivity in older individuals, however, could reflect a compensatory response induced by early pathology (Jagust and Mormino, 2012, Jones et al., 2016, Schultz et al., 2017).

In young adult *APOE*-ε4 carriers, who are highly unlikely to harbor Aβ (Mormino, 2014), increased functional activity in the pDMN and MTL has been seen across AD-relevant cognitive tasks (Dennis et al., 2010, Filippini et al., 2009, Shine et al., 2015). Young *APOE*-ε4 carriers also display greater intrinsic functional connectivity in the DMN than noncarriers (Filippini et al., 2009)—consistent with the view that functional activity differences may reflect increased connectivity. This contrasts with studies in older, cognitively normal *APOE*-ε4 carriers, which typically report decreased functional connectivity (and also activity) in pDMN regions (Sheline et al., 2010).

Extending these studies, we found that college-aged *APOE*-ε4 carriers had increased *structural* connectivity (see below) in the PHCB—the main white matter pathway of the pDMN (Greicius et al., 2009). Specifically, young adult *APOE*-ε4 carriers had lower MD and higher FA than noncarriers. The direction of this effect contrasts with studies in older, cognitively normal *APOE*-ε4 carriers and MCI, where decreased FA (and increased MD) is typically seen (Heise et al., 2014, Villain et al., 2008). Notably, these differences were not seen in a control tract in the temporal lobe—the ILF—which, in contrast to the PHCB, is more affected in semantic dementia than AD (Bejanin et al., 2017). A complementary whole-brain voxelwise analysis also identified no significant differences between carriers and noncarriers outside our key PHCB ROI. Furthermore, to demonstrate that these differences in structural connectivity are linked to heightened pDMN activity in *APOE*-ε4, we correlated interindividual variation in PHCB microstructure with pDMN BOLD response during a scene discrimination task that is sensitive to early cognitive changes in AD (Lee et al., 2006). This multimodal, individual differences approach demonstrated that individuals with the highest pDMN activity during scene discrimination had the highest structural connectivity in the PHCB (lower MD/higher FA), suggesting that individual variation in structural connectivity in the PHCB may drive activity in pDMN, and subsequent vulnerability to Aβ in later life (Buckner et al., 2009, Jagust and Mormino, 2012).

Although group differences in MD and FA most likely reflect an impact of *APOE*-ε4 on some aspect(s) of structural connectivity, we cannot readily determine what these are; variation in these diffusion metrics could arise from multiple, physiologically relevant connectivity properties (e.g., myelination, membrane permeability and/or axon number, diameter, and voxelwise configuration [Jones et al., 2013a, Jones et al., 2013b]). One possibility is that these white matter differences reflect early neuropathology, such as axonal loss (e.g., Shi et al., 2017). The pattern reported here, however, is opposite to that seen typically in older individuals, where studies have reported lower FA and/or higher MD in individuals with AD and MCI (Mito et al., 2018, Rieckmann et al., 2016, but see Racine et al., 2014). Rather, these findings more strongly support an LSV view of AD risk, where early-life, nonpathologically driven structural and functional alterations in specific brain networks may confer risk for later-life AD neuropathology (Jagust and Mormino, 2012).

One possible explanation for these early-life white matter differences is that *APOE*-ε4 carriers and noncarriers may undergo different patterns of white matter maturation. Previous neurodevelopmental studies have highlighted that efficient communication between distributed brain regions may emerge across development via overgrowth and then pruning of redundant axons (Innocenti, 2017, Yeatman et al., 2012). Given recent evidence that the *APOE*-ε4 allele decreases synapse pruning in mice (Chung et al., 2016; see also Wolf et al., 2013, for a review of *APOE* involvement in neurodevelopment), it is tempting to speculate that *APOE*-ε4 carriers may display somewhat reduced or delayed axonal pruning of the late-maturing cingulum during a critical period, such as adolescence (see Yeatman et al., 2012, Yeatman et al., 2014). This could feasibly lead to an “overshoot” in tissue microstructure and concomitant increases in pDMN neural activity. Increased pDMN activity in young adult *APOE*-ε4 carriers (as seen here during scene discrimination) may thus reflect some form of lifelong reduced network efficiency (Jagust and Mormino, 2012; see also; Mesulam, 1999) or flexibility (Westlye et al., 2011), which impacts on the ability of the pDMN to efficiently modulate activity (or functional connectivity with MTL) (Harrison et al., 2016, Westlye et al., 2011) in line with the needs of a particular task. Note, although increased pDMN activity may feasibly confer certain cognitive benefits earlier in life but increase risk of AD-related pathology in later life (i.e., a form of “antagonistic pleiotropy”; O’Donoghue et al., 2018, Rusted et al., 2013, Tuminello and Han, 2011), we found no differences between *APOE*-ε4 carriers and noncarriers on performance measures for the scene odd-one-out task itself (Section 3.4). To date, there is mixed evidence regarding the effect of *APOE*-ε4 on cognitive performance, with studies reporting both beneficial (Alexander et al., 2007, Evans et al., 2014, Mondadori et al., 2007, Rusted et al., 2013, Stening et al., 2016) and deleterious (Bloss et al., 2010, Sinclair et al., 2015, Yasen et al., 2015) effects. While not providing direct support for this view, the observation of increased structural connectivity/functional activity alongside matched performance seems most likely indicative of reduced efficiency.

Critically, the early-life increases in pDMN structural connectivity reported here (i.e., higher FA/lower MD), and concomitant changes in functional activity (Shine et al., 2015), may portend a faster decline in connectivity over the lifespan (Brown et al., 2011, Felsky and Voineskos, 2013) and ultimately lead to early Aβ deposition, which in turn facilitates tau-mediated neurodegeneration (de Haan et al., 2012, Jacobs et al., 2018). For instance, a cross-sectional study, which applied graph theory to measure the network characteristics of dMRI data, found that younger *APOE*-ε4 carriers had greater “local interconnectivity” relative to noncarriers but exhibited a steeper age-related reduction (Brown et al., 2011; see also Felsky and Voineskos, 2013). A potential compensatory later increase in connectivity/activity, in response to accumulating Aβ pathology in early disease stages (Schultz et al., 2017), may result in further increased nodal stress and ultimately network failure (Jones et al., 2016), as reflected by an eventual steep decline in pDMN network integrity (activity/connectivity) (Jagust and Mormino, 2012). Future longitudinal multimodal imaging studies would provide further insights into how *APOE*-ε4 influences white matter microstructure and task-related activity across the lifespan.

Although we observed significant differences for both FA and MD, our reported effects were somewhat stronger for MD, particularly for the tractography analysis. This is consistent with reports that FA shows greater intra-tract variability than MD—that is, tracts do not have a signature FA value that is consistent along the tract length (Yeatman et al., 2012). Future dMRI studies using advanced tract profiling and biophysical modeling would shed further insight into the relationship between *APOE*-ε4 and different aspects of PHCB microstructure (Assaf et al., 2017, Yeatman et al., 2012).

While comparable to several previous studies in the literature (Dennis et al., 2010, Filippini et al., 2009, Oh and Jagust, 2013), the sample size in the present study is relatively modest. As such, interpretative caution is needed, and a replication of these effects in a larger independent sample will be required—particularly where such differences in PHCB microstructure can be assessed across the lifespan (Brown et al., 2011). This issue is partly mitigated by (1) the clear hypothesis-driven approach (Button et al., 2013), (2) applying methods that enhance the robustness and precision of key measures (i.e., HARDI, cardiac gating, CSD; Mackinnon, 2013), and (3) Bayesian analyses showing that our findings are informative and have high evidential value (Dienes, 2014).

Furthermore, although our groups were matched for gender, alongside other key variables (i.e., education, age), our sample was predominantly female. Although relatively underexplored, previous studies have suggested that the effect of *APOE*-ε4 on AD-relevant neuroimaging and neuropathological markers is more pronounced in females (Ungar et al., 2014). Such markers include deposition of both Aβ and tau (Corder et al., 2004), and resting-state functional connectivity within the pDMN (Damoiseaux et al., 2012). Concomitant with this, we saw slightly stronger effects of *APOE*-ε4 carrier status on pDMN structural connectivity when the two male participants were removed. It is possible, therefore, that these functionally relevant differences in PHCB microstructure may be less pronounced in young male carriers. Examining the influence of gender on *APOE*-related brain changes will require larger-scale cohort studies, where the power to detect such interactions is increased.

## 5 Conclusion

To conclude, we have shown that *APOE*-ε4–related increases in pDMN activity (Shine et al., 2015) are linked to indices of structural connectivity in the PHCB—the main white matter conduit linking the pDMN with the MTL (Heilbronner and Haber, 2014). Specifically, *APOE*-ε4 carriers had significantly lower MD, and higher FA, in this pathway—the opposite effect to that seen in cognitively normal and cognitively impaired older *APOE*-ε4 carriers (Felsky and Voineskos, 2013, Mito et al., 2018). By combining dMRI and BOLD task-fMRI measures, we showed that interindividual variation in PHCB microstructure (increased FA/decreased MD) was linked to increased pDMN (and MTL) activity during a scene discrimination task that is affected in AD (Lee et al., 2006). These findings support an LSV model of AD risk, whereby genetically influenced connectivity-associated increases in pDMN activity across the lifespan may confer risk for Aβ accumulation in later life—one of the earliest biomarkers of AD pathology.

## Disclosure

The authors declare no actual or potential conflict of interests.
